# Public sentiments toward COVID-19 vaccines in South African cities: An analysis of Twitter posts

**DOI:** 10.3389/fpubh.2022.987376

**Published:** 2022-08-12

**Authors:** Blessing Ogbuokiri, Ali Ahmadi, Nicola Luigi Bragazzi, Zahra Movahedi Nia, Bruce Mellado, Jianhong Wu, James Orbinski, Ali Asgary, Jude Kong

**Affiliations:** ^1^Africa-Canada Artificial Intelligence and Data Innovation Consortium (ACADIC), York University, Toronto, ON, Canada; ^2^Laboratory for Industrial and Applied Mathematics, York University, Toronto, ON, Canada; ^3^Faculty of Computer Engineering, K.N. Toosi University, Tehran, Iran; ^4^School of Physics, Institute for Collider Particle Physics, University of the Witwatersrand, Johannesburg, South Africa; ^5^Dahdaleh Institute for Global Health Research, York University, Toronto, ON, Canada; ^6^Advanced Disaster, Emergency and Rapid-Response Simulation (ADERSIM), York University, Toronto, ON, Canada

**Keywords:** COVID-19, vaccine, vaccination, sentiment analysis, tweets, South Africa, vaccine hesitancy

## Abstract

Amidst the COVID-19 vaccination, Twitter is one of the most popular platforms for discussions about the COVID-19 vaccination. These types of discussions most times lead to a compromise of public confidence toward the vaccine. The text-based data generated by these discussions are used by researchers to extract topics and perform sentiment analysis at the provincial, country, or continent level without considering the local communities. The aim of this study is to use clustered geo-tagged Twitter posts to inform city-level variations in sentiments toward COVID-19 vaccine-related topics in the three largest South African cities (Cape Town, Durban, and Johannesburg). VADER, an NLP pre-trained model was used to label the Twitter posts according to their sentiments with their associated intensity scores. The outputs were validated using NB (0.68), LR (0.75), SVMs (0.70), DT (0.62), and KNN (0.56) machine learning classification algorithms. The number of new COVID-19 cases significantly positively correlated with the number of Tweets in South Africa (Corr = 0.462, *P* < 0.001). Out of the 10 topics identified from the tweets using the LDA model, two were about the COVID-19 vaccines: uptake and supply, respectively. The intensity of the sentiment score for the two topics was associated with the total number of vaccines administered in South Africa (*P* < 0.001). Discussions regarding the two topics showed higher intensity scores for the neutral sentiment class (*P* = 0.015) than for other sentiment classes. Additionally, the intensity of the discussions on the two topics was associated with the total number of vaccines administered, new cases, deaths, and recoveries across the three cities (*P* < 0.001). The sentiment score for the most discussed topic, vaccine uptake, differed across the three cities, with (*P* = 0.003), (*P* = 0.002), and (*P* < 0.001) for positive, negative, and neutral sentiments classes, respectively. The outcome of this research showed that clustered geo-tagged Twitter posts can be used to better analyse the dynamics in sentiments toward community–based infectious diseases-related discussions, such as COVID-19, Malaria, or Monkeypox. This can provide additional city-level information to health policy in planning and decision-making regarding vaccine hesitancy for future outbreaks.

## 1. Introduction

Despite a few antivirals that have been approved very recently by the US FDA against coronavirus (1), preventive measure(s) against the virus is still very relevant (2, 3). According to World Health Organization (WHO) (4, 5), vaccination is one of the primary preventive measure against the novel coronavirus, in addition to other measures already in place to curb the spread of the virus such as social distancing, the use of face masks, sanitization, and isolation (6). To vaccinate or not to vaccinate has become a very important question facing communities in South Africa and the world at large as the COVID-19 pandemic lasts (7–10). As vaccine uptake across South Africa increases, new cases and deaths because of the COVID-19 virus remain (11–13). The unvaccinated people with serious illness and fatalities are the most admitted as reported by most hospitals in South Africa (12).

However, public bias or sentiments influenced by some religious leaders, social media influencers or legal restrictions, as reflected in most of the anti-vaccination messages on social media platforms (5, 14, 15), may have a significant impact on the progression toward achieving vaccination against COVID-19 in South Africa, especially in the local communities (13, 16). Social Media platforms are applications that enable communication amongst users or groups to interact, share, or reshare information on the Internet using different platforms or devices within the comfort of their homes (5, 17). Information sharing on social media spread very fast even if it is a rumor from an unverified source. The impact of rumors is always dangerous, especially in places where users are not well informed about the subject of discussion (14).

Twitter being one of the most influential social media platforms, has become a good tool for sharing news, information, opinions, and emotions about COVID-19 vaccine-related discussions (6, 14, 18, 19). As Twitter users remain connected while observing COVID-19 restrictions, misinformation, unconfirmed rumors, vaccination, and anti-vaccination messages regarding COVID-19 continue to spread (3, 17, 20, 21). These messages which are mostly text-based spread in the form of users' posts or retweets, without confirming their sources. These types of discussions may have contributed in weakening the confidence level of the public well before they were vaccinated (18, 22, 23). Given a large amount of text-based data from Twitter, a lot of research has leveraged on it to draw insight and make predictions on the users' sentiment of the COVID-19 vaccines at a continent, country, or province level while neglecting the local communities (15, 19, 20, 24).

In this study, we used clustered geo-tagged Twitter posts to inform city-level variations in sentiments toward COVID-19 vaccine-related topics in the three largest South African cities (Cape Town, Durban, and Johannesburg). We started with an analysis of Twitter posts from the South African context from January 2021 to August 2021 to understand the popular topics that are being discussed within the period. Then, an exploration of users' sentiments toward the vaccines and how they inform vaccine uptake was conducted. Finally, we performed a comparison of the popular topics and sentiments across the three cities. The approach used in this research showed that geo-tagged Twitter posts can be used to better analyse the dynamics in sentiments toward community-based infectious diseases-related discussions, such as COVID-19, Malaria, or Monkeypox. This can provide additional city-level information to health policy in planning and decision-making regarding vaccine hesitancy for future outbreaks.

## 2. Materials and methods

### 2.1. Data collection

With an existing Twitter account, we applied for Developer Access and were granted access to Twitter Academic Researcher API which allows for over 10 million tweets per month. Then, we created an application to generate the API credentials (access tokens) from Twitter. The access token was used in Python (v3.6) script to authenticate and establish a connection to the Twitter database. To get geo-tagged vaccine-related tweets, we used the Python script we developed to perform a historical search (archive search) of vaccine related keywords with place country South Africa (ZA) from January 2021 to August 2021. By geo-tagged tweets, we refer to Twitter posts with a know location. These vaccine-related keywords include but are not limited to *vaccine*, *anti*−*vaxxer*, *vaccination*, *AstraZeneca*, *Oxford*−*AstraZeneca*, *IChooseVaccination*, *VaccineToSaveSouthAfrica*, *JohnsonJohnson*, and *pfizer*. The keywords were selected from the trending topic during the period of discussion. A complete list of the keywords used can be found in Section 1.1 of the Supplementary material. The preferred language of the tweet is English.

A total of 45,000 tweets was pulled within the period of discussion using the archive search. Each Tweet contains most of the following features: *TweetText*, *TweetID*, *Create*−*Date*, *RetweetCount*, *ReplyCount*, *LikeCount*, *GeoId*, *GeoCityProvince*, GeoCountry, GeoCoordinate(bbox), and *Location*. Others are *AuthorID*, *UserName*, *Hashtags*, *CreatedAccountAt*, *FollowerCount*, *FollowingCount*, and *TweetCount*.

Additionally, daily statistics of new COVID-19 cases, vaccinations, deaths, and recoveries in Cape Town, Durban, and Johannesburg were used. These statistics were obtained from the South African coronavirus official website (24) and the COVID-19 South Africa dashboard (25) which are primarily updated every day.

### 2.2. Data preprocessing

User tweets contain a lot of information about the data they represent. That means, raw tweets that have not been processed are highly unstructured and contain a lot of redundant information. To overcome these issues, preprocessing of raw tweets have become increasingly paramount. In this study, the tweets, date created, location, country, and geocoordinate (bbox) (26) were extracted from the dataset into a dataframe using pandas (v1.2.4) (27).

**Figure 1:**
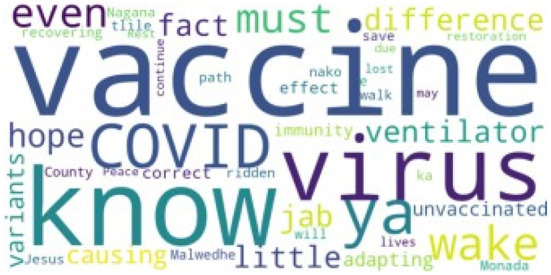
The most frequently used words of our dataset.

We prepared the tweets for Natural Language Processing (NLP) by removing the URLs, duplicate tweets, tweets with incomplete information, punctuations, special and non-alphabetical characters, emojis, non-English words, and stopwords using the tweets-preprocessor toolkit (v0.6.0) (28), Natural Language Toolkit (NLTK; v3.6.2) (29), and Spacy2 toolkit (v3.2) (30, 31). The Spacy2 toolkit was used to perform lowercase and tokenization of the tweets. This process reduced the tweets in the dataset to 25,000. The word-cloud generated from the dataset shows vaccine as one of the most frequent words (see Figure 1).

### 2.3. Sentiment analysis

**Figure 2:**
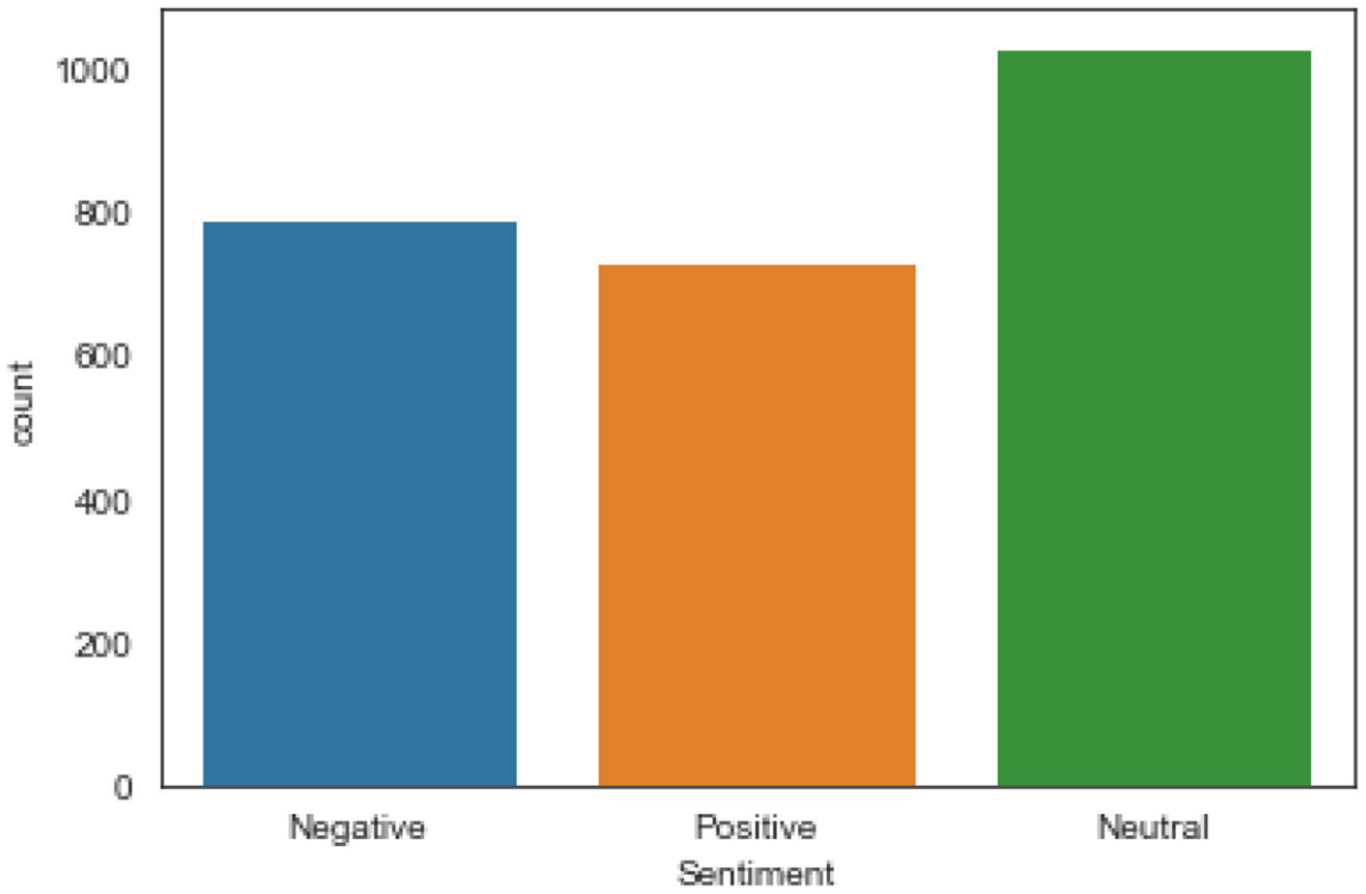
Distribution of manually labeled tweets.

We applied VADER (Valence Aware Dictionary for Sentiment Reasoning) (32, 33) available in the NLTK package to the tweets to get compound scores and assign labels to the tweets. The compound score was used to determine when the label (positive, negative, or neutral) can be assigned to a tweet. A compound score ≥0.5, <0, and *x*, where *x* satisfies the inequality 0.5 > *x* ≥ 0 are assigned the label positive, negative, and neutral, respectively. Further, we randomly selected 2,500 (10%) of the tweets and manually labeled them as positive, negative, or neutral (see Figure 2) to ensure VADER accurately labeled the tweets.

The dataset was divided into two parts, namely, training set (0.80) and test set (0.20) which was fitted into five different machine learning classification algorithms for evaluation and prediction. The algorithms are Naive Bayes (NB) (34), Logistic Regression (LR) (35, 36), Support Vector Machines (SVM) (36, 37), Decision Tree (DT) (38), and K-Nearest Neighbors (KNN) (39). We chose these algorithms because they have been tested to perform well with classification and prediction with text based dataset.

### 2.4. Topic modeling

The Latent Dirichlet Allocation (LDA) (40) model was used for the topic modeling of the dataset through the Gensim (v4.0.1) (41) package in Python. The LDA was used because it is assumed to be one of the popular models for this type of analysis, is easy to use, and has been successfully used and implemented in recent studies such as (18) and (9, 15). The LDA models were created for 1–10 topics to optimize the number of topics. Jaccard similarity (42, 43) and coherence measure (44) tests were administered and calculated across the topics. The Jaccard similarity (*sklearn*.*metrics*.*jaccard*_*s*_*core*) package in Python was used for a test of the uniqueness of the topics while the coherence (*gensim*.*models*.*coherencemodel*) package using the c_v (45) option in Python was used for the measure of the degree of similarity between high scoring words in the topic.

### 2.5. Test statistics

The trends in time between vaccine-related tweets and new COVID-19 cases in South Africa were compared using the Granger causality test (46) from the *statsmodels*.*tsa*.*stattools* import *grangercausalitytests* package in Python. The correlation coefficient was calculated using the Pearson correlation from the *scipy*.*stats*.*pearsonr* package in Python. Further, the intensity of the sentiments of each vaccine-related topic was also compared using the Mann-Whitney *U* test (47) from the *scipy*.*stats*.*mannwhitneyu* package in Python.

Similarly, the time series trends for the intensity of the vaccine-related topics for each of Cape Town, Durban, and Johannesburg were compared to the total vaccinations, new cases, deaths, and recoveries using the Granger causality test. The Mann-Whitney *U*-test was used to compare the distribution of the sentiment intensity for each vaccine-related topic for each city.

Finally, the sentiment intensity distribution for the vaccine trending topic across the three cities was compared using the Kruskal Wallis *H*-test (48) from the *scipy*.*stats*.*kruskal* package in Python.

## 3. Results

### 3.1. Our dataset in South African context

The Supplementary Figure 1 shows the summary statistics of our dataset in the South African context with time. As shown in Supplementary Figure 1, there is upward growth in the number of tweets in the first, second, and third weeks of January and February. However, there are some levels of consistency in growth in the number of vaccine-related tweets for every other week of the month.

**Figure 3:**
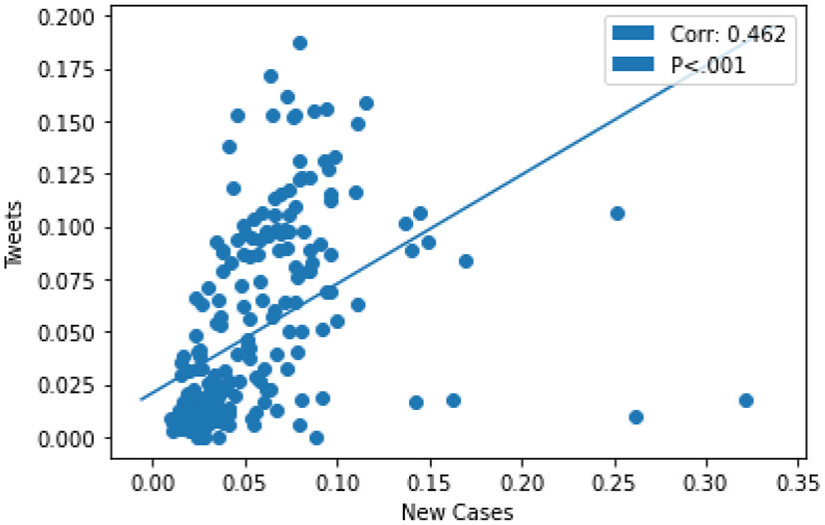
The number of new COVID-19 cases significantly positively correlated with the number of Tweets.

The trend in the growth of the daily tweets and daily COVID-19 cases proved to be consistent with time. For instance, the upward growth in the number of daily tweets correlated with growth in the number of daily new COVID-19 cases for January and July. Similarly, the decline in the numbers of daily tweets and a daily number of cases demonstrated a similar trend over time (see Supplementary Figure 2). Therefore, the number of new COVID-19 cases significantly positively correlated with the number of Tweets (corr = 0.462, *p* < 0.001, and 95% CI) as shown in Figure 3.

### 3.2. Sentiment in the South African context

The five machine learning algorithms were used to build models that classified the tweets as positive, negative, and neutral. The COVID-19 dataset comprising 25,000 tweets were classified. Accuracy, precision, recall, F1-Score, Receiver Operating Characteristic (ROC), and Area Under the curve (AUC) metrics were taken as performance measure for the quality of the multi classification output. The summary of the models performance output is summarized in Table 1.

**Table 1:** Tweet sentiment model classification performance.

**SN**	**Algorithm**	**Accuracy (%)**	**Average precision (%)**	**Average recall (%)**	**Average F1-score (%)**	**ROC (%)**	**Average AUC (%)**
1	NB	0.68	0.66	0.62	0.63	0.79	0.79
2	LR	0.75	0.74	0.70	0.72	0.88	0.88
3	SVMs	0.70	0.73	0.61	0.63	0.86	0.86
4	DT	0.62	0.58	0.56	0.57	0.67	0.67
5	KNN	0.56	0.56	0.40	0.37	0.62	0.62

**Figure 4:**
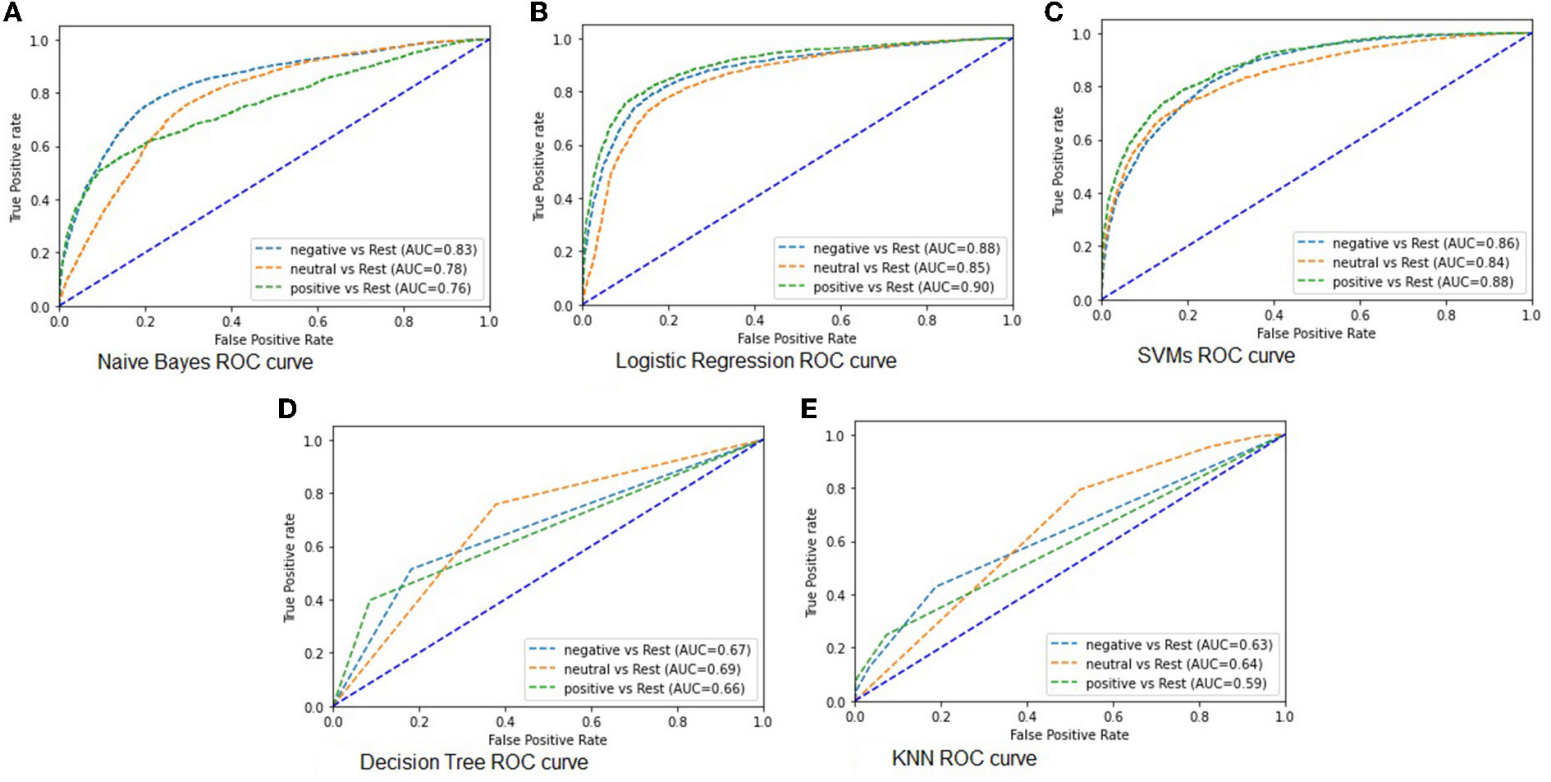
**(A–E)** ROC–AUC for different models of tweet sentiments classification.

As shown in Table 1, there is a clear difference in the accuracy scores of the models. The LR model demonstrated best performance in classifying the tweets sentiments as positive, negative, and neutral with an accuracy score of 75% and ROC-AUC scores of 88%. Similarly, KNN demonstrated to have performed weakly in classifying the sentiments with an accuracy scores of 56% and ROC-AUC score of 62%. The above analysis in Table 1 shows that these models have the ability to classify tweets according to their sentiments. However, the LR model proved to be best fit for this type of classification problem given all indicators. One such indicator is that the 25,000 tweets generated a large feature set that was suitable for the LR higher performance. Next, we visualized ROC metric to evaluate the quality of the multi classification output, together with the AUC (see Figure 4).

As shown in Figure 4, we want to ascertain how well the models classified each sentiment class. The ROC curve shows the sensitivity also called true positive rate against specificity. The specificity is also called true negative rate. The true positive rate is the probability of the model to accurately predict the properly labeled sentiment from the tweets. While the true negative rate is the probability of the models to accurately predict the mislabeled sentiments from the tweets. The more the curve aligns toward the upper left corner of the plot, the better the model does at classifying the tweets into various sentiment classes. The LR model does well in classifying the tweets into various sentiment classes, see Figure 4B followed by SVMs model in Figure 4C. Unlike the NB model with an average performance in classification of the tweet sentiments (Figure 4A), the DT and KNN models performed poorly in classifying the tweets into different sentiment classes, see Figures 4D,E, respectively. The AUC was used to ascertain how much of the plot is located under the curve. If the AUC score is closer to 1, then, the model is assumed to have performed well. Therefore, the LR model demonstrated to have performed better with a large feature set and multiclass prediction.

Since the LR model performed better than the other models, understanding the features that influenced the sentiment classification of the tweets is necessary. We used ELI5 (49), an interpretable machine learning model to visualize the top twenty features in their order of importance for the logistic regression model. Table 2 shows the weight and features of the top twenty words that influenced the sentiment classes of the tweets as classified by the logistic regression model.

**Table 2:** The LR model feature interpretation using ELI5.

**SN**	**Positive**		**Negative**		**Neutral**	
	**Weight**	**Feature**	**Weight**	**Feature**	**Weight**	**Feature**
1	+3.215	Best	+2.837	Died	+1.944	Bias
2	+2.855	Positive	+2.194	Death	−1.284	Fear
3	+2.826	Wow	+2.190	Worse	−1.284	Trust
4	+2.785	Happy	+2.143	Crisis	−1.286	Crisis
5	+2.659	Love	+2.098	Fuck	−1.305	Fraud
6	+2.584	Great	+2.050	Killed	−1.321	Worst
7	+2.511	Free	+1.939	Suspended	−1.333	ffs
8	+2.484	Encourage	+1.929	Fake	−1.343	Great
9	+2.212	Loved	+1.905	Pain	−1.359	Negative
10	+2.068	Amazing	+1.876	Dangerous	−1.432	Amazing
11	+2.011	Beautiful	+1.873	Hate	−1.438	Bad
12	+1.914	Ensure	+1.825	Kills	−1.455	Sorry
13	+1.899	Dear	+1.818	Worst	−1.486	Steal
14	+1.879	Rich	+1.812	Conspiracy	−1.524	Celebrating
15	+1.859	Happily	+1.799	Scam	−1.613	Kill
16	+1.764	Safety	+1.754	Hell	−1.645	Encourage
17	+1.755	Peace	+1.748	Cancer	−1.652	Killing
18	+1.752	Luck	−1.888	Protected	−1.712	Conspiracy
19	+1.750	Grateful	−1.889	Loved	−1.739	Love
20	−1.758	Died	−1.968	Best	−1.904	Wow

**Figure 5:**
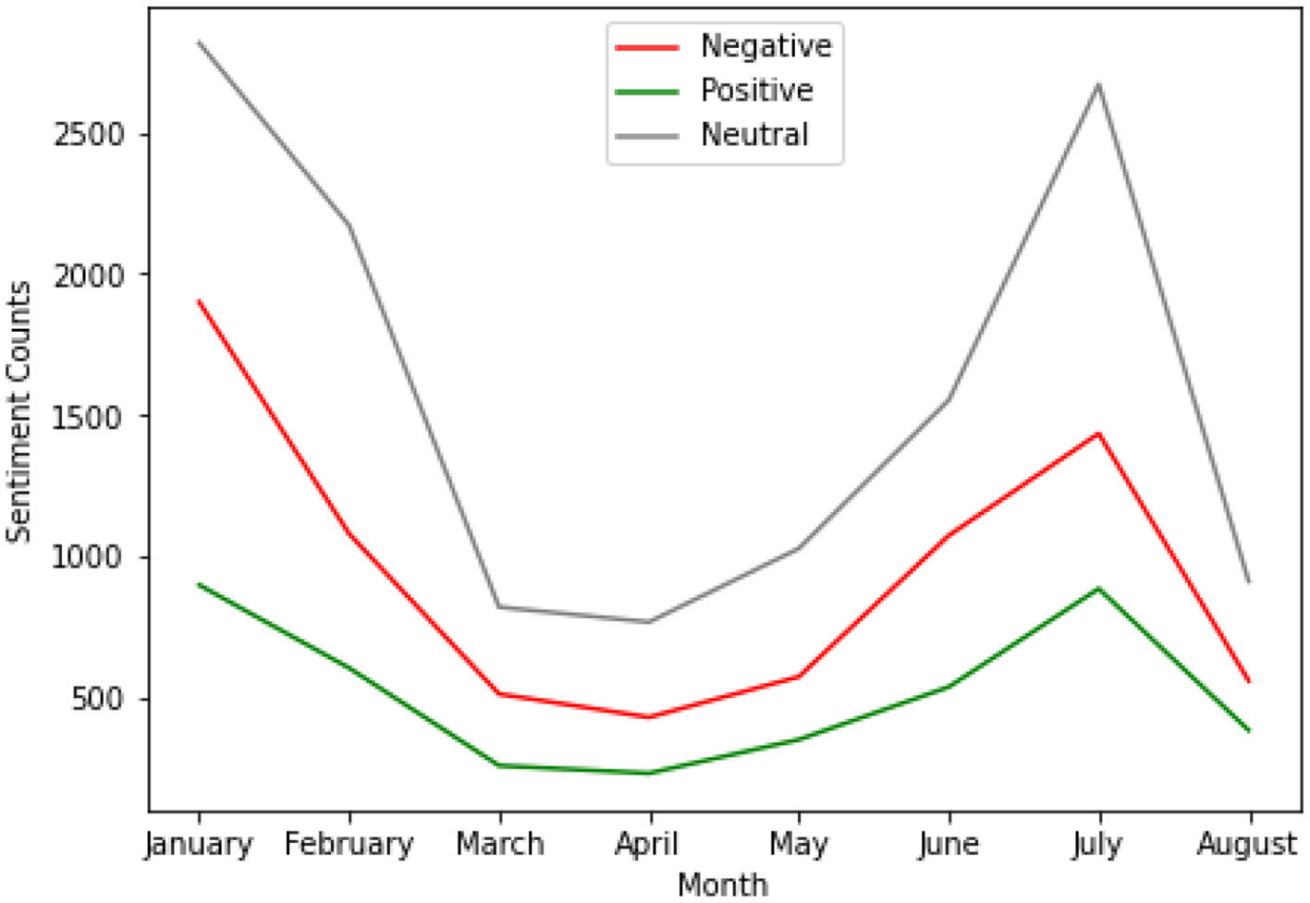
Tweets sentiments classes in South Africa from January to August 2021.

Figure 5 summaries the distribution of the classified tweets sentiments with time. As shown in Figure 5, there is growth in sentiment with time for all the sentiment classes. In January and July, the neutral sentiment class maintained an upward growth followed by the negative and positive sentiment classes respectively. Additionally, there is a decline in growth in April and August for all the sentiment classes. The difference in sentiment classes between January and the other months is statistically significant (*p* < 0.001 and 95% CI).

### 3.3. Identifying COVID-19 vaccine topics in the South African context

After the application of the LDA model on the tweets, 45 topics were generated. Some of which are the same and incoherent by observation. We applied the Jaccard similarity test to ascertain the uniqueness of each topic. The Jaccard similarity counts the number of similar words in two topics and divides it by the total number of words from the two topics combined. If the Jaccard similarity value is 1 it shows that the topics are the same and 0 otherwise. We considered topics whose Jaccard similarity value is <0.5. However, to be sure that the topics identified are semantically acceptable, we performed a coherence measure test on the topics. High coherence measure value shows that the topic could be meaningful, hence we chose topics that yielded high coherence values.

This process reduced the generated topics to 10 unique topics. The topics are vaccine uptake (topic 1), social distancing (topic 2), xenophobic attack (topic 3), travel restrictions (topic 4), alcohol ban (topic 5), religion (topic 6), sports (topic 7), border closure (topic 8), politics (topic 9), and vaccine supply (topic 10). Two topics were identified and considered to be relevant to this study, which are, vaccine uptake (topic 1) and vaccine supply (topic 10), respectively. The first 10 top-scoring representative words for topics 1 and 10 and their possible interpretations are shown in Table 3.

**Table 3:** Selected LDA generated topics and their interpretations.

**Topic number**	**Representative word**	**Possible interpretations**
1	Jab, vaccine, pfizer, get, first, dose, jampj, second, got, done	Got my first jab. Done with my first pfizer vaccine jab. Received a second dose of the johnson & johnson vaccine
2	Vaccine, covid, people, govern, countries, money, world, supply, sa, virus	This topic focuses on the need for the South African government to pay for the supply of more COVID-19 vaccine.

**Figure 6:**
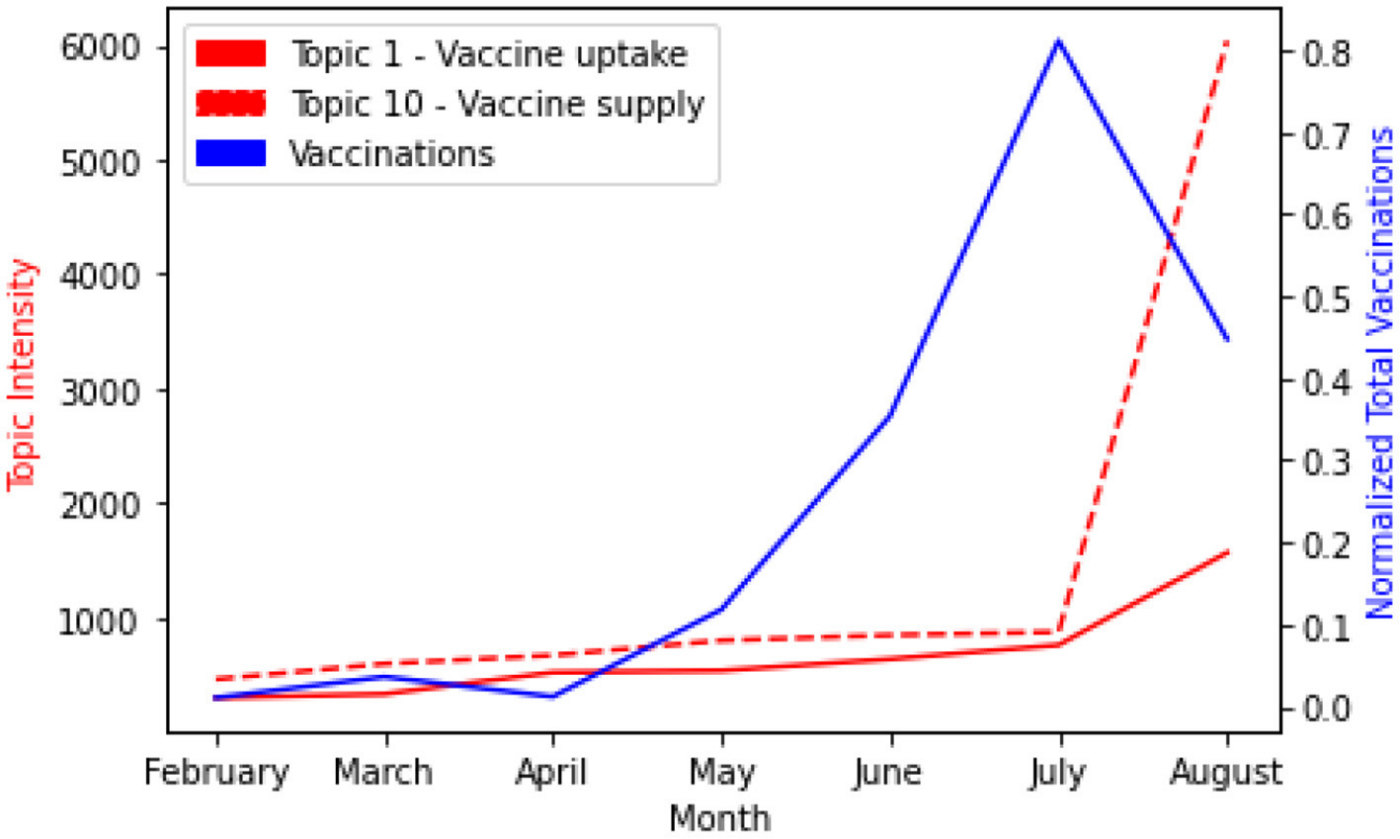
Comparing vaccine-related discussions with the number of people vaccinated.

Next, we compared the level of the vaccine-related discussions to the number of people vaccinated (see Figure 6). We observed that the intensity of topics 1 (red line) and 10 (dashed red line) started increasing almost at the same pace from February to June with topic 10 slightly higher. We ignored January because the rollout of vaccines started from February in South African as shown in the data available to us [see (24)]. However, in July, the two topics showed higher intensity than the other months. Topic 10 started to grow upward from July to August. Further, comparing this outcome with the total number of people vaccinated (blue line) within the period. We observed that, while the vaccine-related discussions increase from February to July, the total number of vaccinations also increased. July to August showed a sharp decrease in vaccination while the vaccine-related discussions increased. An evaluation of the impact of vaccine-related discussions on the total vaccinations using the Granger test for causality showed that an increase in vaccine-related discussions correlates with the number of people vaccinated, *P* = 0.004 for the two topics.

Further, we present the distribution and compare the differences in sentiment intensity scores between the two topics. We observed that the sentiment intensity score for both topics had significantly higher scores for neutral class (*P* = 0.015) than the negative sentiment (*P* = 0.024) and the positive sentiment (*P* = 0.035) classes, respectively (see Supplementary Figure 3).

**Figure 7:**
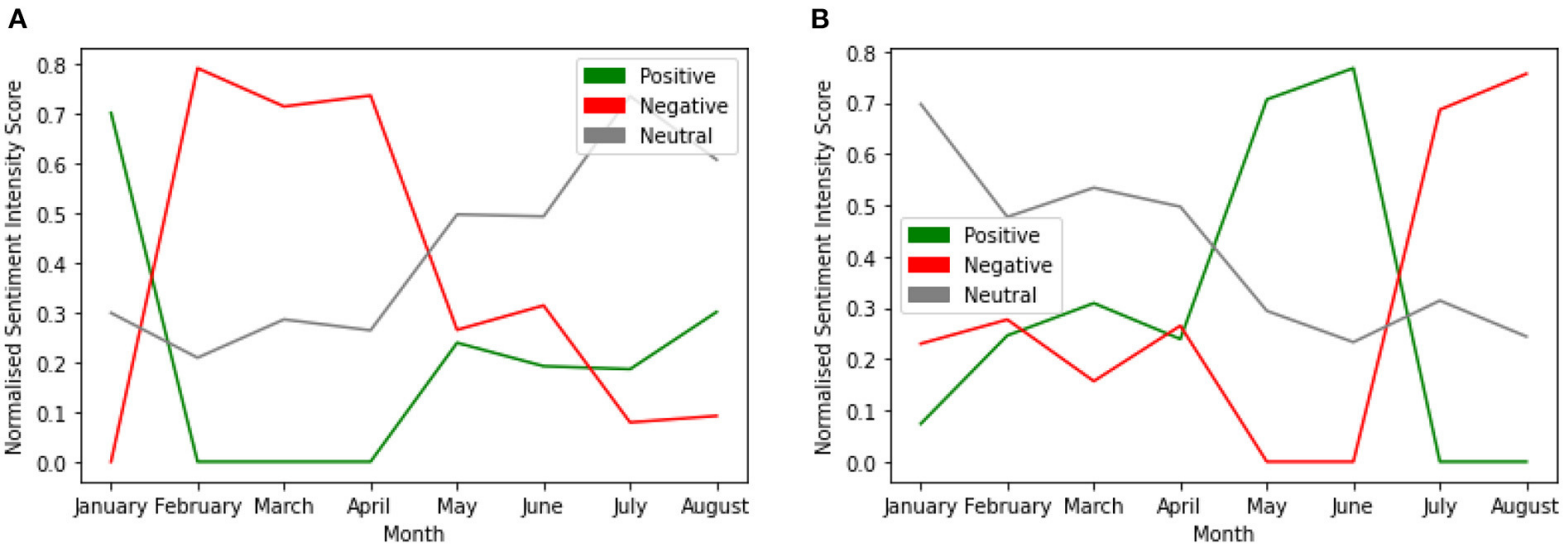
Trends in sentiment intensity with time for vaccine related discussions. **(A)** Trend in sentiment intensity with Time for Topic 1. **(B)** Trend in Sentiment intensity with Time for Topic 10.

We further investigated the differences in trends in sentiment intensity scores between the two topics with time (see Figure 7). In January, the sentiment intensity for negative sentiment started to trend upward and downward for the positive sentiment. Both flattened between February and April. There was a sharp decline between April and May for the negative sentiment intensity and an increase for the positive sentiment within the period. However, the intensity for the neutral sentiment trended upward with time for Topic 1. Trend in sentiment intensity with Time for Topic 1 is shown in Figure 7A.

Similarly, for topic 10, the sentiment intensity started to trend upward for the positive and negative sentiments in January. While the positive sentiment intensity score continued to trend upward until June when it started to decline, the negative sentiment intensity score trended downward until June when it trended upward. Additionally, the neutral sentiment intensity continued to trend downward with time for topic 10. Trend in Sentiment intensity with Time for Topic 10 is shown in Figure 7B.

### 3.4. City-level analysis of vaccine discussions

To investigate the city-level analysis of vaccine-related discussions, we selected tweets for three major cities namely, Cape Town (*n* = 2,484), Durban (*n* = 1,020), and Johannesburg (*n* = 2,898) from the South African dataset that we preprocessed and labeled. We chose these cities because they are the largest cities by population in South Africa (50). Supplementary Figure 4 summarizes the distribution of the selected tweets according to the location.

We also present the distribution of the sentiments of the preprocessed tweets according to each selected city as shown in Supplementary Figure 4. The reason for this is to enable us to identify the city-specific discussions and to analyze the intensity of their sentiments. We applied the LDA model on the preprocessed tweets at city-level. Then, the Jaccard similarity and coherence tests were applied to these topics. These processes enabled us to identify two unique topics that are relevant to our research across each city. These topics are vaccine uptake (Topic 1) and vaccine supply (Topic 3).

#### 3.4.1. Cape town specific analysis

**Figure 8:**
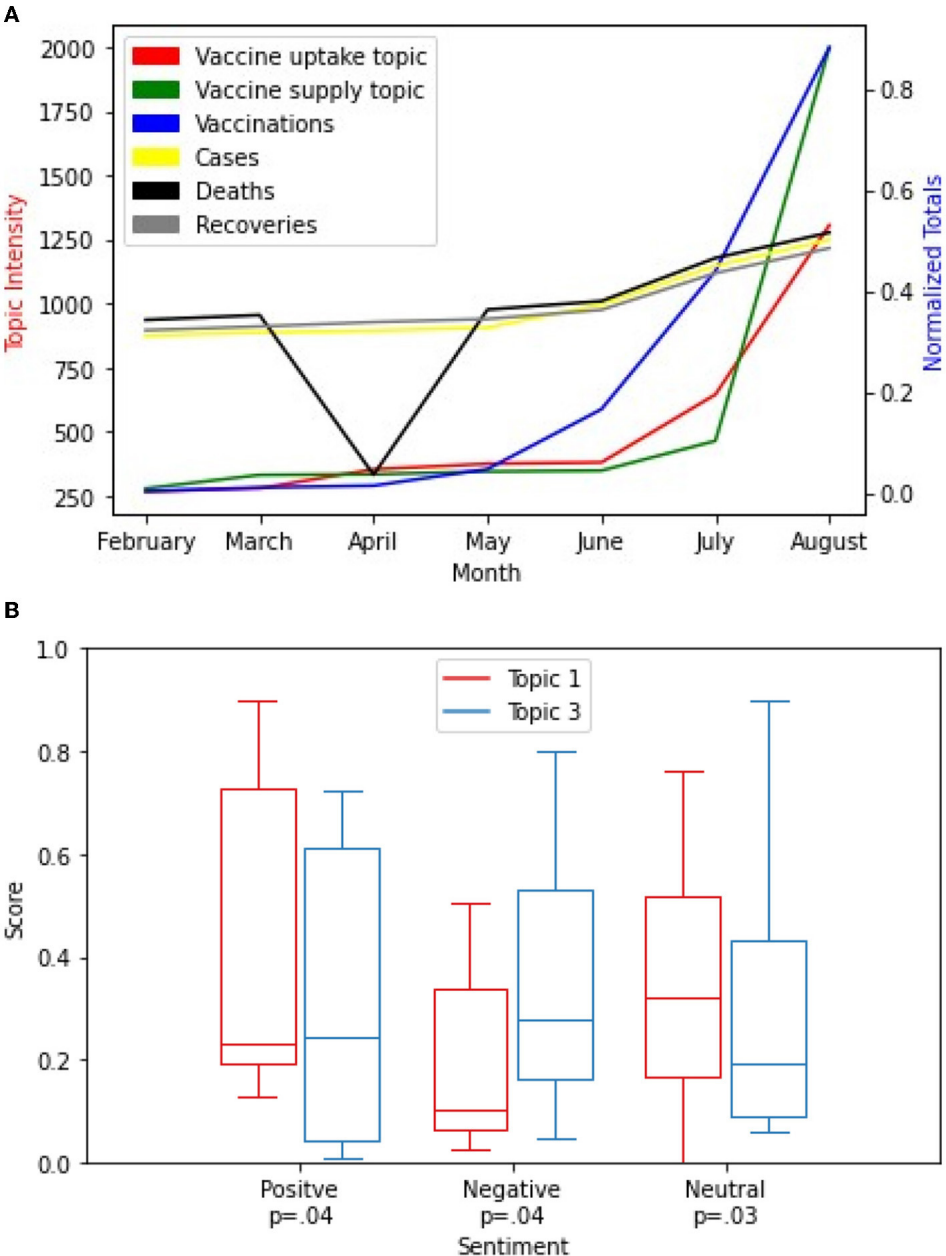
**(A,B)** Cape Town City Analysis.

We compared the level of the vaccine-related discussions in Cape Town to the number of people vaccinated, new cases, deaths, and recoveries in the Western Cape Province (see Figure 8A). This is because there is no South African city-level COVID-19 Data (24, 25) accessible to us at the time of this research. We chose to compare the Western Cape province data to the Cape Town city vaccine-related discussions because Cape Town is the largest city in the Western Cape province (50).

As the intensity score for topics 1 and 3 trends upward, total vaccinations also increased from February to August. The evaluation of the impact of vaccine-related discussions on the total vaccinations in the Western Cape province using the Granger test for causality showed a strong statistically significant correlation *P* < 0.001 for the two topics. However, as the intensity of topics 1 and 3 increase, the number of new cases and recoveries also increase but at a slow pace from February to August. There is a sharp decline in the number of COVID-19 related deaths in April than other months. The impact of vaccine-related discussions on the new cases, deaths, and recoveries showed a weak correlation (*P* = 0.07; see Figure 8A).

The summary of the distribution of the sentiment intensity scores is shown in Figure 8B. A comparison of the differences in sentiment intensity scores between the two topic 1 and topic 3 in Cape Town demonstrated a higher sentiment intensity score for both topics for the neutral sentiment (*P* = 0.03) class than the positive sentiment class (*P* = 0.04) and the negative sentiment class (*P* = 0.04), respectively.

#### 3.4.2. Durban specific analysis

**Figure 9:**
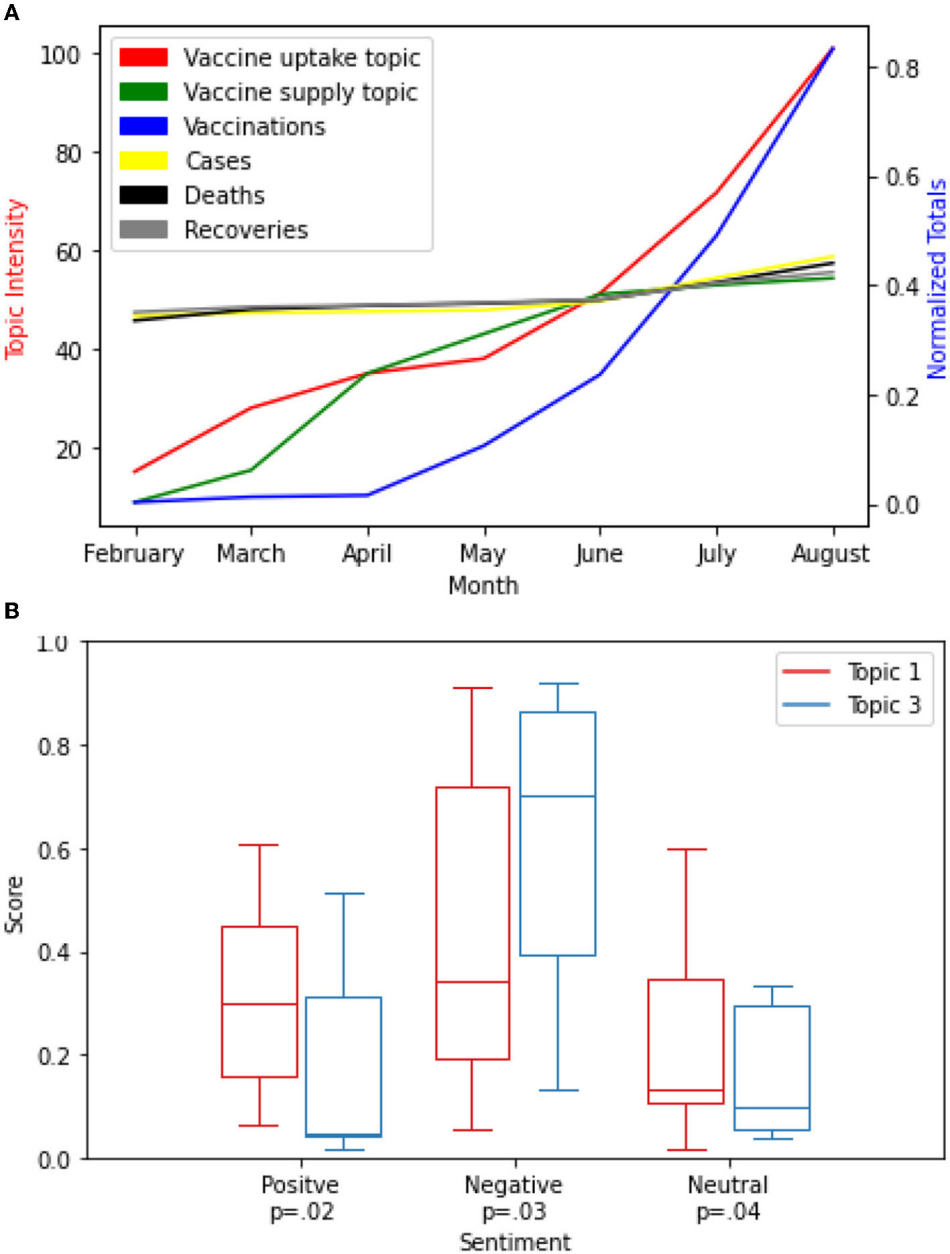
**(A,B)** Durban City Analysis.

While the intensity of topics 1 and 3 trends upward, respectively, the total vaccinations increased almost at the same pace especially from February to August 2021. The evaluation of the impact of vaccine-related discussions on the total vaccinations in the Kwazulu-Natal province using the Granger test for causality showed a strong statistically significant correlation *P* < 0.001 for the two topics. However, as the intensity of the two topics increased, the number of new cases, deaths, and recoveries almost flattened from February to August 2021. The evaluation of the impact of vaccine-related discussions on the new cases, deaths, and recoveries showed a weak correlation (*P* = 0.07; see Figure 9A).

Next, we present the distribution and compare the differences in sentiment intensity scores between the two topics 1 and 3 for Durban city. We observed that the sentiment intensity score for both topics demonstrated higher scores for the negative sentiment class (*P* = 0.03) than the positive sentiment class (*P* = 0.02) and the neutral sentiment class (*P* = 0.04), respectively (see Figure 9B).

#### 3.4.3. Johannesburg specific analysis

**Figure 10:**
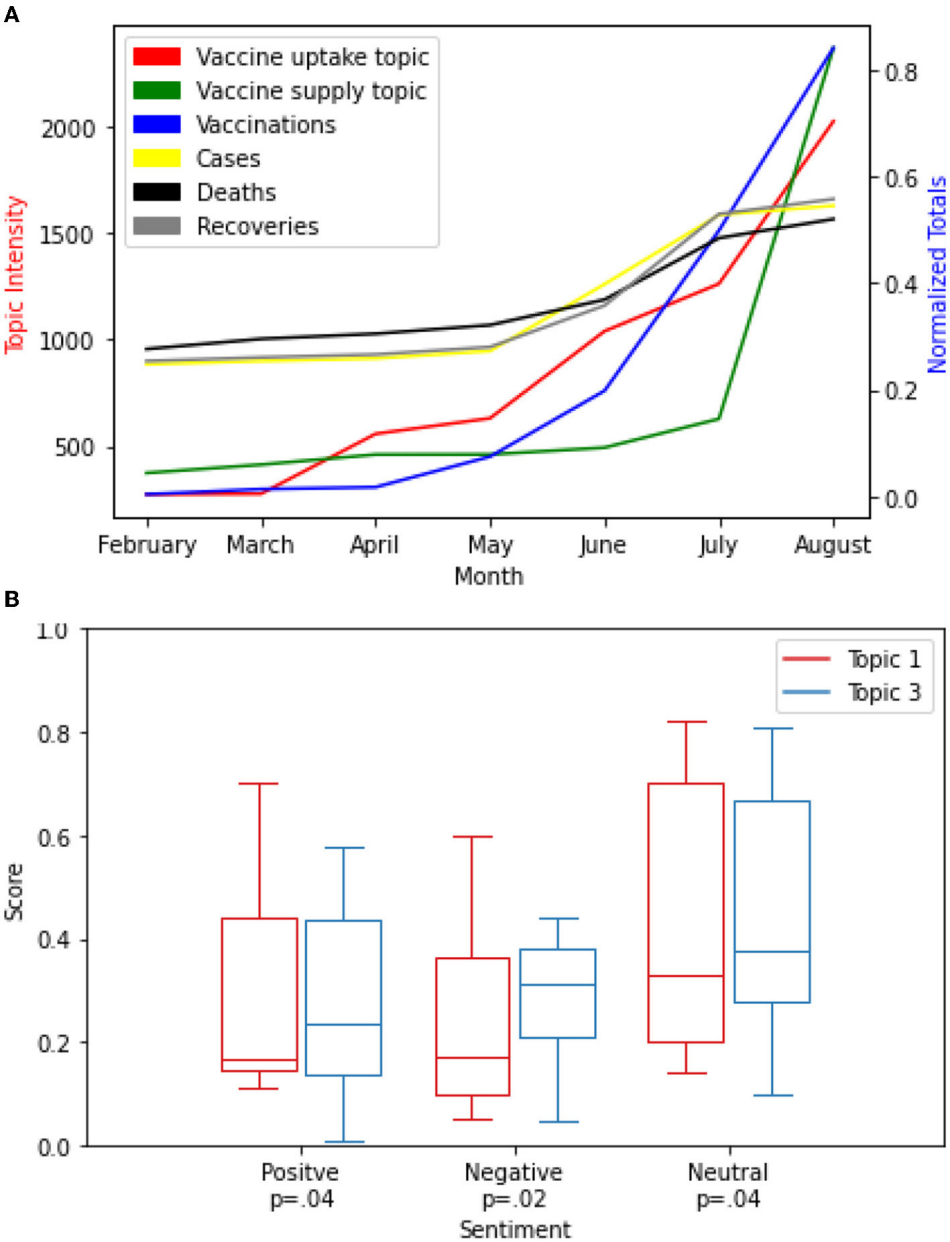
**(A,B)** Johannesburg City Analysis.

Unlike Cape Town and Durban, Johannesburg showed a strong correlation on the impact of the vaccine-related discussions on new cases, deaths, and recoveries in the Gauteng province (*P* = 0.03) with time. Similarly, as the intensity score for topics 1 and 3 trends upward, total vaccinations also increased from February to August. The evaluation of the impact of vaccine-related discussions on the total vaccinations showed a strong statistically significant correlation *P* < 0.001 for the two topics (see Figure 10A).

Further, we presented the distribution of the sentiments for each topic and compared the differences in sentiment intensity scores between the two topics as well. We observed that the sentiment intensity scores for both topics demonstrated higher scores for the neutral sentiment (*P* = 0.04) than the positive sentiment (*P* = 0.04) and the negative sentiment (*P* = 0.02), respectively (see Figure 10B).

#### 3.4.4. Comparison across cities

**Figure 11:**
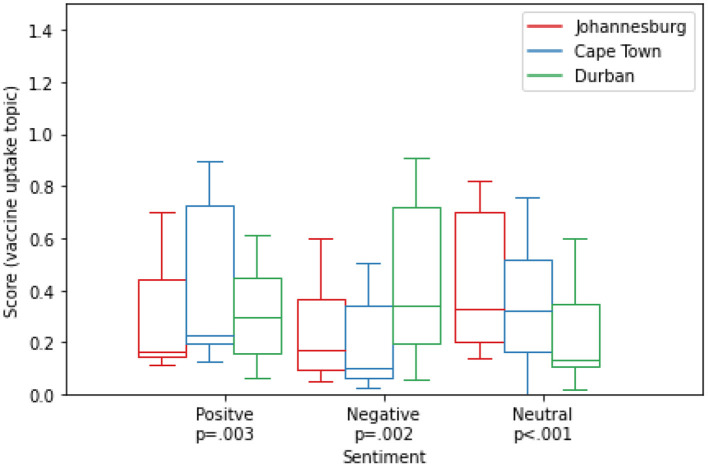
Distribution and Comparison of Sentiment intensity scores for vaccine uptake across cities using the Kruskal-Wallis *H*-test.

Sentiment toward vaccine uptake deferred across cities (see Figure 11). Cape Town demonstrated a higher intensity score for positive sentiment class than Durban and Johannesburg. Durban demonstrated a higher negative sentiment intensity score than Cape Town and Johannesburg. Similarly, Johannesburg demonstrated a higher neutral sentiment than Durban and Cape Town. There is a statistically significant neutral sentiment class (*p* < 0.001) for the vaccine uptake topic across the cities than the negative sentiment class (*p* = 0.002), and positive sentiment class (*p* = 0.003), respectively. The Word clouds for vaccine uptake topic across the three cities are shown in Supplementary Figure 6.

Next, we present the uncommon challenges encountered during this research.

### 3.5. Limitations

The dataset used for this research only reflects the opinion of Twitter users whose geolocation was South Africa from January 2021 to August 2021. South Africa with a population of about 60 million people has only 15% online adults who use Twitter, and of the aged 18–35 (51). Therefore, this research does not, at large, provide the opinion of the people of South Africa regarding COVID-19 vaccines. However, this research only provided an insightful prediction of vaccine-related discussions from our dataset which was also used to complement exiting vaccination data to support policy making in managing vaccine hesitancy.

It is also relevant to state here that most NLTK for sentiment analysis techniques do not have the capacity to properly label figurative language, such as sarcasm. However, since the approach we used was able to label and score a large amount of the tweets in our dataset and was verified with the manual labeling of randomly selected (10%) of the tweets, in addition to the 70% accuracy achieved with the SVM classification algorithm, we assume it was able to deal with the noise generated by this obvious challenge. Finally, since our data generation ended in August 2021, the suggested area of further studies could be the generation and use of a larger dataset up to a recent date.

### 3.6. Ethical considerations

The study was approved by Twitter and access was granted to the Twitter academic researcher API which was used to retrieve the tweets. All retrieved tweets are in the public domain and are publicly available. However, the authors strictly followed the highest ethical principles in handling the personal information of Twitter users, as such, all personal information was removed.

## 4. Discussions

We have used the Twitter API to generate and process a dataset of vaccine-related Twitter posts in South Africa from January 2021 to August 2021. We observed a decline in daily tweets and new cases between March 2021 and April 2021. This could be attributed to the effect of the Xenophobic attack (52) and the Zuma unrest (53) that took place in South Africa during this period. Our result showed that the number of new COVID-19 cases in South Africa significantly positively correlated with the number of Tweets.

The LDA topic modeling approach was used to generate topics on South Africa Twitter dataset we processed. We identified 10 topics, namely, vaccine uptake, social distancing, Xenophobic attack, travel restrictions, alcohol ban, religion, sports, border closer, politics, and vaccine supply. The vaccine uptake and vaccine supply were the most dominant topics. This approach could be likened to be similar but not the same as the study in (15). In (15), the LDA topic modeling and aspect-based sentiment analysis (ABSA) were used on Twitter data at a continental level, North America in particular. The LDA was used to identify different topics relating to COVID-19 in the USA and Canada, respectively. According to the study in (15), travel and border restrictions were the most discussed topics in February 2020 and were later overtaken by discussions about physical distancing with time. Contrary to the VADER and SVM we used in our study for sentiment analysis, ABSA was used to identify various sentiments related to the overall outbreak, anti-Asian racism and misinformation, and positive occurrences related to physical distancing.

Further, our study showed that an increase in vaccine-related discussions correlated with the number of people vaccinated for the two dominant topics we identified. We observed that the sentiment intensity score for both topics had significantly higher scores for the neutral class than the other classes. This could be attributed to the fact that a lot of people at this time may be indecisive about taking the vaccine. As a result, a lot of vaccines expired without being administered to people, this is similar to what is seen in other African countries like Nigeria, Mozambique, Zimbabwe, Botswana, Eswatini, Angola, Democratic Republic of Congo, etc. (54, 55), where a lot of vaccines are said to have expired without being administered to people, despite the fact that a low percentage of their populations are vaccinated. This type of information could be helpful to public health agencies to understand public concerns of Twitter users toward vaccine hesitancy especially in communities where the acceptance rate is low.

The increase in intensity score of the negative sentiment class for the vaccine uptake topic from February 2021 to April 2021 seems to have a slight effect on the number of vaccinations, especially in April 2021, this could be the result of the rumors and conspiracy theorist concerning the side effect of the vaccines (14) and the presidential address that vaccination is not compulsory at that time, as such, a lot of people seem to be hesitant in taking the vaccine because of one reason or another (24). In July 2021, there was a lot of continuous media and physical sensitization campaigns and awareness of the need to be vaccinated by the health agencies in South Africa (24, 25), hence, as the number of vaccinations continued to increase, the sentiment intensity scores for the vaccine uptake topic also increased for the neutral and positive sentiment classes. In August 2021, our result appeared to behave differently. While the intensity scores for the neutral and positive sentiment classes for the vaccine supply topic decreased the number of vaccinations also decreased. Furthermore, analysis on the three selected cities, Cape Town, Durban, and Johannesburg, showed different sentiment intensity scores on the two topics within the period of discussion. This suggests that city—specific policy can be helpful in addressing the sentiment toward vaccine hesitancy.

For example, Cape Town showed a strong significant correlation of the impact of the upward increase in the intensity scores for both topics to the total vaccinations from February 2021 to August 2021. There was a weak correlation of the impact of the vaccine uptake and supply topics to new cases, deaths, and recoveries. Cape Town also demonstrated a higher sentiment intensity score for both topics for neutral sentiment class than other sentiment classes.

However, in Durban, the impact of vaccine-related discussions on the total vaccinations showed a strong correlation for the two topics. The impact of vaccine-related discussions on the new cases, deaths, and recoveries demonstrated a weak correlation from February 2021 to August 2021. Additionally, the sentiment intensity score for both topics demonstrated higher scores for the negative sentiment than the other sentiment classes.

From February to August, Johannesburg demonstrated a strong correlation on the impact of the vaccine-related discussions to new cases, deaths, and recoveries for both topics. The sentiment intensity scores for both topics demonstrated higher scores for the neutral sentiment than the other classes.

Finally, a comparison across cities for the most trending topic, vaccine uptake, showed that Cape Town demonstrated a higher intensity score for positive sentiment class, while Durban and Johannesburg demonstrated higher negative and neutral sentiments classes, respectively. There is a statistically significant neutral sentiment class for the vaccine uptake topic across the cities. Our analysis showed that Twitters posts can be used to better understand the city-specific sentiment on vaccines related topics. Given that this approach is fast and less expensive, health policymakers could adopt this approach in monitoring citizens' responses to related policies. For example, the study in (56) showed how sentiment analysis could be used to understand public perceptions of policies in Italy. This was very helpful in the accountability and responsiveness of policymakers. Similarly, the study in (18) showed engagement on Reddit correlated with COVID-19 cases and vaccination rates in Canadian cities. This showed that discussion on social media can serve as predictors for real-world statistics.

## 5. Conclusion

In this research, Twitter posts containing daily updates of location-based COVID-19 vaccine-related tweets were used to generate topics and understand the sentiments around the topics. Trending topics regarding the vaccine discussions were identified at local levels. The impact of the sentiment of these discussions was identified and related to the vaccinations, new COVID-19 cases, deaths, and recoveries at the local levels.

These go further to show that clustered geo-tagged Twitter posts can be used to better analyse the sentiments toward COVID-19 vaccines at the local level. Our results, therefore, suggest that clustered geo-tagged Twitter posts can be used to better analyse the dynamics in sentiments toward community-based infectious diseases-related discussions, such as COVID-19, Malaria, or Monkeypox. This can provide additional city-level information to health policy in complementing existing data for planning and decision-making, especially in managing vaccine hesitancy.

## Data availability statement

The dataset used for this study can be found in the online repository at: https://www.kaggle.com/datasets/ogbuokiriblessing/tweetdatasa.

## Author contributions

BO contributed to the conception, design, analysis, draft, and final revision of the manuscript. ZM contributed to data collection. NB contributed to the manuscript editorial revision. AAh, BM, JW, JO, and AAs contributed to the supervision of the design analysis. JK contributed to supervision of the design analysis and manuscript editorial revision. All authors contributed to the article and approved the submitted version.

## Funding

This research was funded by Canada's International Development Research Centre (IDRC) and Swedish International Development Cooperation Agency (SIDA) (Grant No. 109559-001).

## Conflict of interest

The authors declare that the research was conducted in the absence of any commercial or financial relationships that could be construed as a potential conflict of interest.

## Publisher's note

All claims expressed in this article are solely those of the authors and do not necessarily represent those of their affiliated organizations, or those of the publisher, the editors and the reviewers. Any product that may be evaluated in this article, or claim that may be made by its manufacturer, is not guaranteed or endorsed by the publisher.
