# Supplementary material for: Public sentiments toward COVID-19 vaccines in South African cities: An analysis of Twitter posts
Source: Front Public Health. 2022 Aug 12;10:987376. doi: 10.3389/fpubh.2022.987376 (PMC9412204; doi:10.3389/fpubh.2022.987376)
Supplement: Supplementary file 1 [file Data_Sheet_1.PDF]

Supplementary Material

1 SUPPLEMENTARY FIGURES

Below are list of supplementary figure for the manuscript

1.1 Code Snippet: Search Keywords

```
Oxford-AstraZeneca OR AstraZeneca OR JohnsonJohnson OR Vaccine OR
BioNTech OR anti-vaccine OR jab OR Vaccination OR Covax OR
Vaccine Rollout OR Sputnik OR VaccineToSaveSouthAfrica OR
IChooseVaccination OR TeachersVaccine OR AstraZeneca vaccine OR
Pfizer OR J & J OR Johonson & Johnson OR Moderna OR VaccinesWork
OR VacciNation OR Vaccine OR Steriod OR COVIDvaccine OR covax OR
VaccineEquity OR VaccineReady OR Jab OR PfizerGang OR Scamdemic OR
Plandemic OR Scaredemic OR COVID-19 OR coronavirus OR SARS-CoV-2
OR anti-vaxxers OR jab OR Pfizer, BioNTech OR JJ Vaccine OR
JohnsonJohnson Vaccine OR Vaccine Rollout OR J & J OR Sputnik
OR COVAX OR CoronaVac
```

1.2 Figures

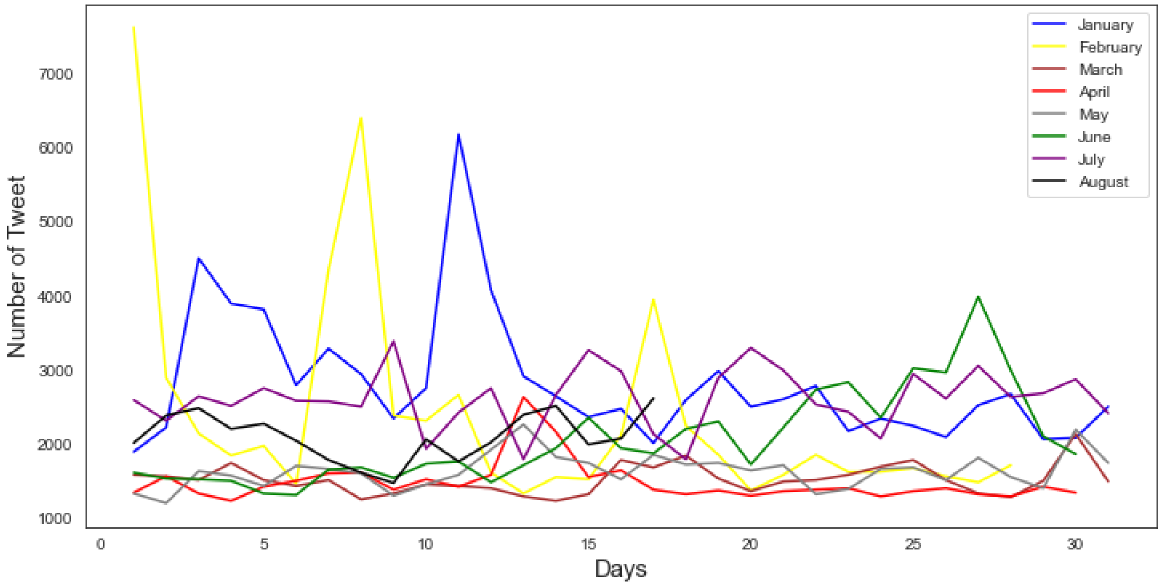

Figure S1. South Africa vaccine-related Tweets from January to August 2021

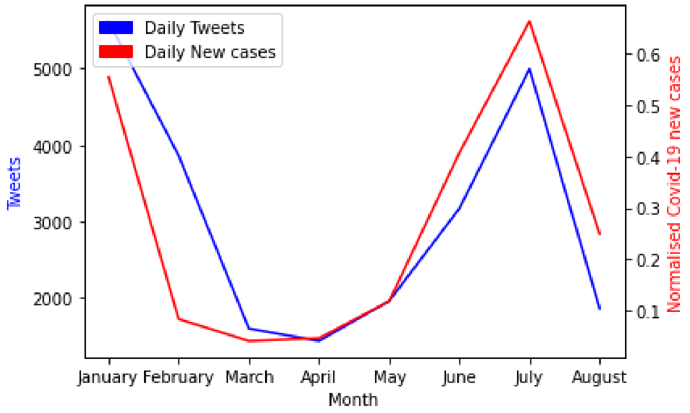

Figure S2. Number of new COVID-19 cases in South Africa and the number of daily Tweets

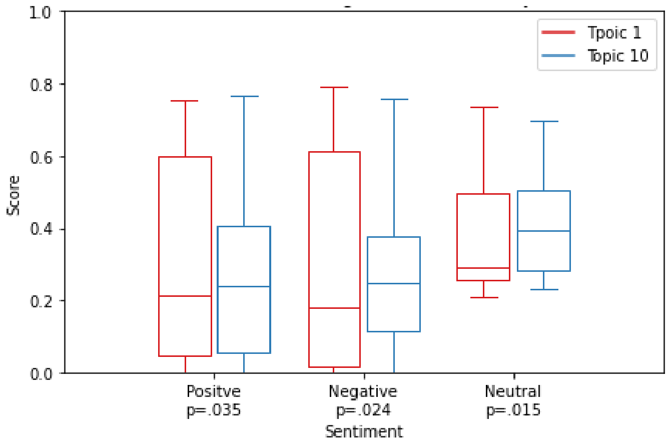

Figure S3. Distribution and comparison of sentiment intensity scores for vaccine-related discussions in South Africa using the Mann–Whitney U test.

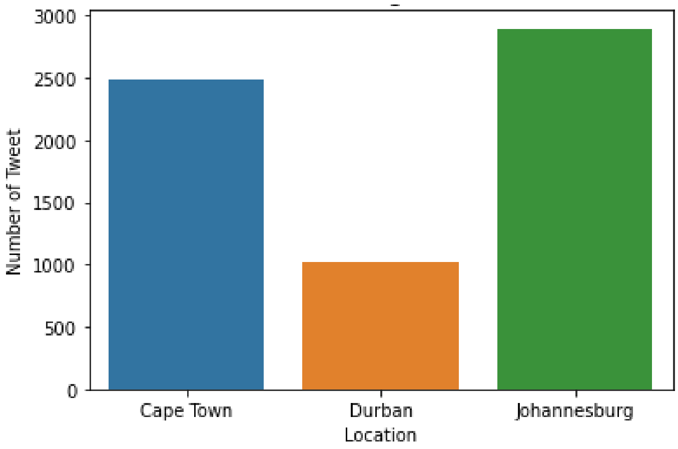

Figure S4. South Africa vaccine-related tweets according to selected location.

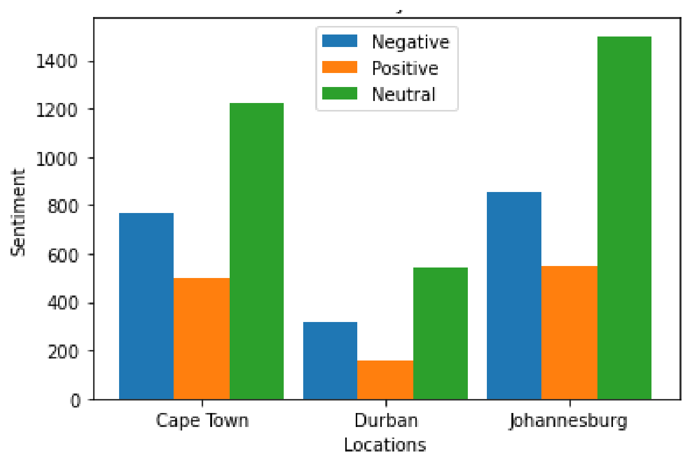

Figure S5. City-Level COVID-19 vaccine-related tweet sentiments classification.

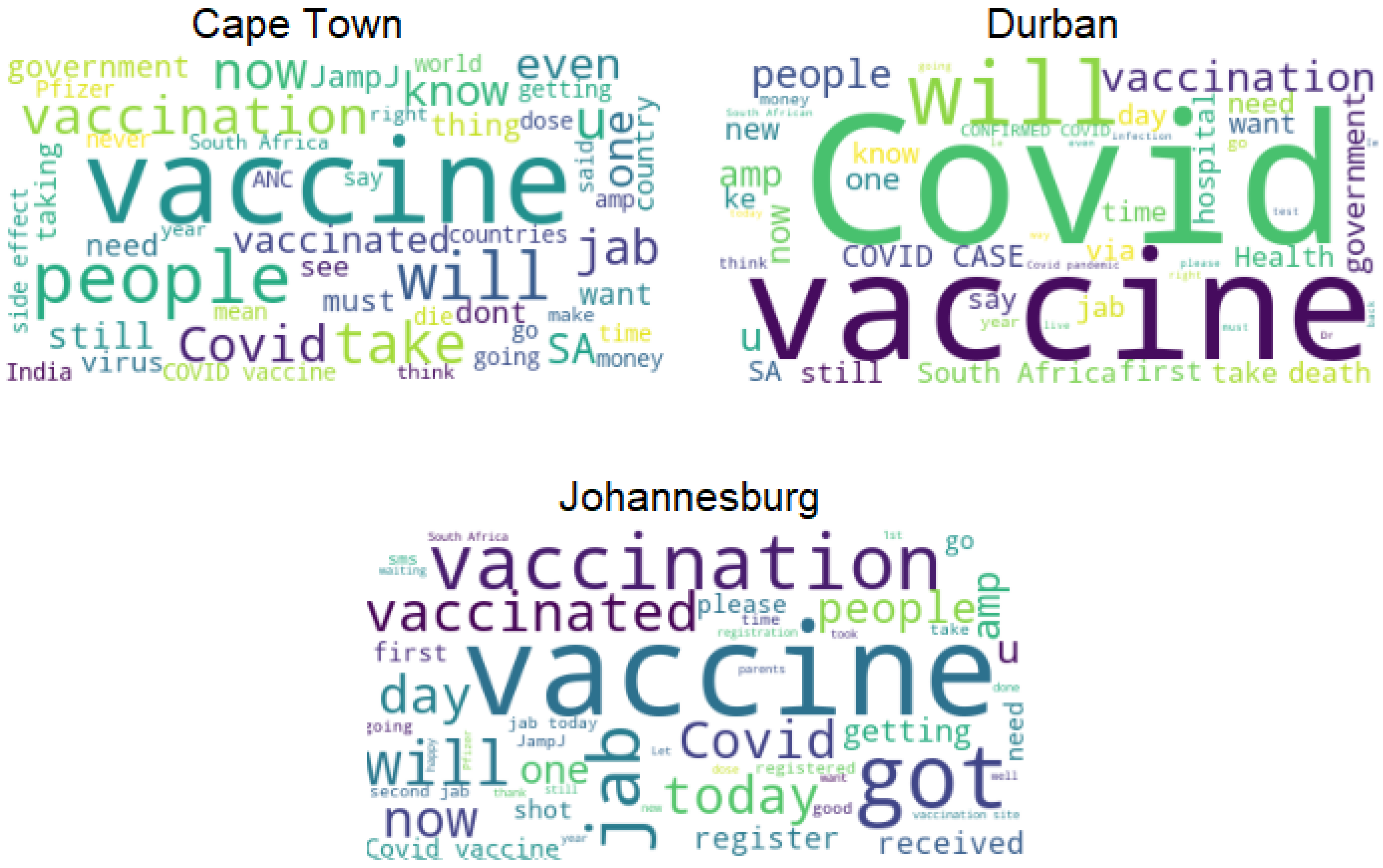

Figure S6. City-Level word cloud for vaccine uptake topic.
